# Better Board Education for Better Leadership and Management in the Health Sectors of Low and Middle Income Countries

**DOI:** 10.3389/fpubh.2019.00067

**Published:** 2019-04-04

**Authors:** Godfrey Sikipa, Egbe Osifo-Dawodu, Gilbert Kokwaro, James A. Rice

**Affiliations:** ^1^CompreHealth, Harare, Zimbabwe; ^2^Partner Anadach Consulting, Lagos, Nigeria; ^3^Strathmore University School of Business, Nairobi, Kenya; ^4^Gallagher Integrated, Minneapolis, MN, United States

**Keywords:** governance, boards, hospitals, leadership, governing bodies

## Abstract

Board member education must be elevated within the curricula of leadership development programming in Low and Middle Income Countries (LMICs) across the globe. When properly trained and supported, the community, business, and health sector leaders serving on these boards can create the conditions within which those who deliver and manage health services are more likely to successfully achieve the mission of their organizations. The importance of incorporating education for governing body members into health sector leadership development programming, and three strategies for board development, are defined in in this article.

## Introduction

Good board work is essential to establish and oversee: policies, the work of healthcare providers and managers needed for the vitality of health sector organizations, and the wise use of funds allocated into the health sectors of Low and Middle Income Countries (LMICs)^1^. Unfortunately, boards and good governance are not well-understood, and often overlooked, in the design, development and management of high performing health sectors in LMICs.

If LMIC health system leaders and educators want stronger health systems and greater health outcomes, they need to be prepared to invest in smarter governance. This includes targeted investments into special programs for the development of the community leaders serving on boards that hire, fire, and hold to account the leaders and managers of health sector organizations, agencies, and programs.

The three key board development strategies described here are:

**Strategy 1: Discovery**: Map and assess the current governance gaps, philosophies and structures within each LMIC health system and each major healthcare organization.
**Strategy 2: Design**: Explore and embrace modern methods for the education, training, and onboarding of board members, using multi-media learning systems and advanced pedagogical techniques.
**Strategy 3: Development**: Implement case based learning programs, materials, and experiences that parallel new education tools and techniques used in executive development.

Before describing these strategies, the article describes the rationale for educating boards and offers a framework for health systems governance that defines content needed for new board development programming in LMICs.

## Understanding the Value of Good Boards and Good Governance

Whether in non-governmental organizations (NGOs), civil society organizations (CSOs)^2^, for-profit private sectors, and facilities run under a Public Private Partnership (PPP) arrangements or decentralized/autonomous organizations of ministries of health, people who lead, manage, or deliver health services benefit when governing bodies and governance decision-making processes are wise and ethical^3^. This is particularly true for low-resourced health systems in low and middle income countries, which are the focus of this article^4^. These governing bodies make decisions about policy, plans, and rules of collective action. When good governance is evident, the members of governing bodies wield power and resources to define, promote, protect, and achieve the health mission of the organization, be it a program within an institution or an entire country.

Good governing bodies offer health sector leaders 10 major health system benefits:

1. They improve rapport and engagement with and support from the community, and enhance our understanding of the health needs of people and communities for business planning and market planning;
2. They expand political influence with local and regional politicians to strengthen our access to needed resources (human, financial, technical, regulatory);
3. They leverage their members' experience and ideas to help develop better plans to expand equitable access to health services;
4. They encourage our leaders to improve our accountability to implement plans and improve our performance for the many constituencies and stakeholders;
5. They help support oversight, accountability, and professional growth for the chief executive officer (CEO), senior management team, and senior clinical leaders in numerous areas. These areas include: clinical expertise including medicine and nursing; health promotion; business expertise; finance; legal matters; marketing; process improvement; total quality improvement; public health; and epidemiology. They also encompass six sigma^5^ thinking and tools; supply-chain management; and change management in turbulent times and with scarce resources;
6. They shield the CEO from pressures from politicians, health workers, staff, vendors, and unions to make inappropriate changes;
7. They foster an objective view of our strategic plans and tactical initiatives by posing challenging questions about their meaning and importance;
8. They bring new and objective perspectives on our problem definitions and problem resolutions;
9. They support the pursuit of philanthropy, grants, funding and/or government backing needed to achieve our mission;
10. They serve as a sounding board to clarify and make plans, strategies, and resource investments more effective.

## Characteristics of LMIC Boards

A nation's health sector is composed of many types of organizations, all of which are likely to have “boards” variously called:

◦ Board of directors
◦ Governing body
◦ Governing council
◦ Board of trustees
◦ Board of commissioners
◦ Board of governors
◦ Board of overseers
◦ Health Committees

While the role and scope of authority of these bodies can vary widely, the engagement of these governing bodies with multiple stakeholders from their communities can represent a positive force for popular capitalism, democracy and economic well-being.

A study for the African Union estimated that over one million people are serving in over 170,000 governing bodies in the health sectors of their 54 member states^6^. Many of those serving on boards do not have formal education that prepares them for leadership roles, nor are they familiar with the operations of healthcare system. Educating the board thus becomes essential to enable the institution they govern to realize the benefits of strong board leadership.

## Framework for Education

**Figure 1:**
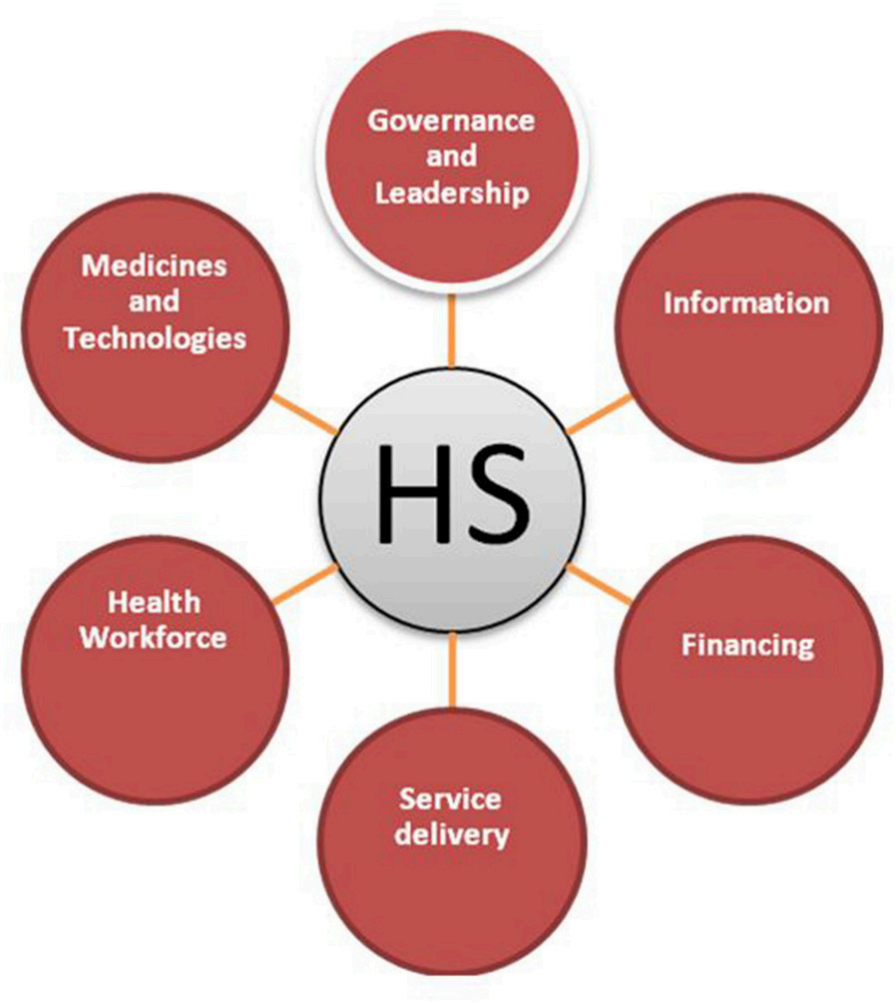
Health system pillars.

For health services organizations, the focus of this collective action is to strengthen all pillars of health systems (Figure 1) in order to expand access to health services^7^. This leads to better and more sustained health outcomes^8^.

A well-performing health system is one that achieves sustained health outcomes through continuous improvement of these six inter-related health system functions:^9^ human resources for health; health finance; health governance; health information; medical products, vaccines, and technologies; and service delivery. Education programs for boards must help board members better understand each of these functions, and what is required to structure and manage them effectively.

A review of USAID funded health sector board development programs in LMICs suggests that good board work consists of mastering and continuously improving these four key practices:

- Setting strategic direction and objectives for an organization;
- Making policies, laws, rules, regulations, or decisions;
- Raising and deploying resources to accomplish the organization's mission, strategic goals, and objectives;
- Overseeing the work of the organization to achieve its mission.

The governing body seeks the best ways to achieve their strategic goals and objectives and enhance the long-term vitality of the organization so it can pursue its mission.

In order to foster good governance for health, people who govern, governing bodies, health sector leaders, and managers at all levels in low- and middle-income countries must become more knowledgeable about good board work. This would include new governing body organizational forms and practices of governing for health.

## Board Obstacles to Good Governance

Health sector managers must therefore master new concepts, strategies, and processes for effective health system board work. This mastery is challenging due to certain obstacles to good governance

Five common obstacles to good board work found in LMICs include:

◦ Boards are not given adequate authority to govern.
◦ Boards are unable to recruit talented and ethical people to serve in the board and its committees.
◦ Board members do not understand their roles and responsibilities.
◦ Boards do not receive support (information, orientation, education and staff help) from managers to successfully engage in wise decision-making.
◦ Boards do not commit to continuously evaluate and refine their governance processes and practices.

Those involved with the education of board and administrative leaders can overcome these obstacles by: (a) being ever vigilant to identify and commit to remove each obstacle, and (b) understanding and applying the positive activities and practices described below.

## Three Strategies for Good Board Education

To overcome these challenges, and the above cited board work obstacles, intentional educational programming investments are essential within the three strategies of Discovery, Design, and Development.

### Strategy 1: Discovery: Map and Assess the Current Governance Gaps, Philosophies and Structures Within Each LMIC Health System

Educators and health sector policy leaders should understand the numbers and types of governing bodies that already exist in their nation's health sector. They should annually survey these governing bodies to develop a written profile of the numbers and types of people serving on these governing bodies. Basic demographic information should be tracked to ensure that gaps in needed diversity and experience can be identified, and action plans designed to address such gaps. Polling to define topics judged necessary for high performance board decision-making can also be included in such surveys. This information serves as background for curricula design and recruiting board members into multi-media learning opportunities and materials.

### Strategy 2: Design: Explore and Embrace Modern Methods for the Education, Training and Onboarding of Board Members Within Multi-Media Learning Systems

The authors' work to conduct board education programs; support the onboarding of new board members; and the design of distance and face-to-face learning methods suggest board member development curricula needs to address these design features:

The Curricula should incorporate content and case studies such as those developed in the USAID “Leaders Who Govern” text, see: https://www.msh.org/resources/leaders-who-govern.

The topics addressed in this comprehensive text include:

1. Role Confusion The general role of governing bodies
2. Composition and Competencies
3. Use of Subgroups
4. Culture to Empower Workers What Is Organizational Culture?
5. Context Constraints
6. Organization Types and Levels
7. Deciding on the Need to Establish a Governing Body: The pros and cons of governing bodies
8. Value and Creation of Terms of Reference for Governing Bodies The value of terms of reference for governing bodies
9. Motivation and Measurement of Performance
10. Clear Processes and Practices
11. Culture of Accountability
12. Stakeholder Engagement
13. Strategy Development
14. Stewardship of Resources
15. Continuous Improvement
16. Management Oversight
17. Member Recruitment
18. Member Orientation and Education
19. Strategic Thinking and Planning
20. Resource Mobilization
21. Quality Assurance
22. Human Resources Development
23. Governance Self-Assessments
24. Communication Plans and Strategies
25. Effective Meetings
26. Themed Meeting Calendar
27. Use of Information
28. Culture of Celebration
29. Governance in Pharmaceutical Systems Managing access to essential medicines

Content for the curricula will vary in the types of curricula and learning methods but essential topics are shown in this exhibit:

**Table:** 

**Common curricula and content**	**Teaching methods and instructors**
Functions of board Board composition Board onboarding and development Organization of health system Quality care and patient experiences Health economic & financing Functions of management Human resources Stakeholder engagement CEO performance development Population and public health Board self-assessment Health care statistics Program evaluation	Board competency based content in the UK national health service are maintained here: https://www.leadershipacademy.nhs.uk/resources/healthy-nhs-board/ Case studies (See Strategy 3 below) Handbooks and guides on each topic See https://www.msh.org/resources/governance-guides-and-handbooks •Classroom learning in ministry of health training centers •Webinars •Speakers in board meetings from ministry of health and the organization's executive team or medical staff •Board strategic planning and education retreats •Study tours to other boards and regions •Attendance at public health association, hospital, and medical society associations Internet based materials can be secured from the WHO, The World Bank, OECD, USAID, DFID, and US Board Development organizations like BoardSource, see: https://boardsource.org/about-boardsource/

### Strategy 3: Development: Implement Case Based Learning Programs, Materials, and Experiences that Parallel New Executive Education Tools and Techniques

◦ As most board members are volunteers, and not necessarily experienced in health systems leadership, management and clinical affairs, learning experiences should be provided in short, targeted executive education style and case study focused programming. A case study from South Africa is here: https://www.researchgate.net/publication/320379417__The__state__of__public__hospital__governance__and__management__in__a__South__African__hospital__A__case__study
◦ The governance of various styles of health sector organizations, from community or district level health councils to national hospital boards, requires different content, and curricula. The learning materials should therefore be focused on the unique needs of each type of organization, such as in the handbooks and guides developed by Management Sciences for Health in their USAID supported project, see: https://www.msh.org/resources/governance-guides-and-handbooks
◦ Low cost distance learning modules have also been made available under this USAID program, see https://www.globalhealthlearning.org/course/governance-and-health-101.

This article seeks to address these strategic governance competency focused challenges and opportunities for leaders and managers working in the health sectors of low and middle income countries. A curriculum focused on seven infrastructure factors of good governing bodies, and the suggested methods and content for teaching boards about these factors was prepared by Management Sciences for Health and is available on-line, see: http://www.msh.org/sites/msh.org/files/msh_ehandbook_complete.pdf.

Great board leaders—both men and women—come in many shapes, sizes, and ages. They come to work representing a variety of backgrounds, experiences, nationalities, languages, cultures, and attitudes, and with a range of knowledge, skills, and competencies. To fully maximize these characteristics, you will find it increasingly necessary and valuable to work with, for, and inside many types and forms of governing bodies. The principles in this article also apply to most types of organizations in wealthier countries and to sectors beyond health. They can also be used in organizations that purchase, finance, or regulate health services.

The work of effective leaders has been previously described in the book *Managers Who Lead*^10^ Leaders and managers here are generally defined to be the men and women who develop, lead, or manage not only health sector–related organizations, but also boards, councils, or commissions dedicated to sustainable health systems strengthening. Roles may also include: Regional/Provincial Medical/Health Directors/Officers; Regional/Provincial/Nursing Officers; District Medical/Nursing Officers. These persons must realize that they have a role to play in health sector governance and it is part of their responsibility or duties to facilitate the work of health governance boards/structures.

Good governance enables those who lead, manage, and deliver health services to be more effective and efficient by:

1. Establishing policies, plans, and procedures that remove obstacles for leaders to do their work;
2. Encouraging leaders to be more successful in supporting the governing body to accomplish the essential governing practices of:
  - cultivating accountability
  - engaging stakeholders
  - setting a shared strategic direction
  - stewarding resources responsibly
  - continuously improving the four practices above;
3. Making available the resources—political, human, technological, and financial—that leaders and health care professionals need to do their work;
4. Expecting, encouraging, and empowering leaders and managers to strive for service delivery that meets or exceeds standards of excellence;
5. Celebrating the organization's journey toward stronger health systems and better health outcomes.

The practices cultivated in board education programming should also be shaped by work to define and live within essential principles for good governance advocated by these international organizations: The Global Fund^11^, World Health Organization^12^, World Bank^13^, Organization for Economic Co-operation and Development (OECD)^14^, and the Center for Healthcare Governance of the American Hospital Association^15^. Good governance is also taken to be key to the achievement of Sustainable Development Goal 3: Ensure healthy lives and promote well-being for all at all ages^16^.

## Conclusion

Countries, health organizations, and health managers that want to achieve sustainable gains in health system performance must master good governance principles, processes and practices. This mastery is more likely to be accomplished throughout all levels of the health sectors in low and middle income countries of Asia, Africa, Europe, and Latin America when well-trained boards are in place and healthcare executives, as well as board members, work together to incorporate training about good governance into recruiting and nurturing their leadership. LMIC leaders need to not only have good educational programs, but in many Low and Middle Income Countries, as well as Developed Countries, board members should be selected-not just because they are wealthy or powerful members of the community. Each member should also commit to learning about the business responsibilities of health sector organizations and to participate in ongoing education. The task of the management is then to provide education that is meaningful, well-structured, appropriate for the audience of board members, and offered on a regular and consistent basis.

## Author Contributions

All authors listed have made a substantial, direct and intellectual contribution to the work, and approved it for publication.

### Conflict of Interest Statement

GS is employed as a principal of Compre Health Services, an education and advisory services organization based in Zimbabwe. EO-D is a principal of Anadach Group, an education and advisory services organization based in Nigeria. JR is a principal of a consulting services firm based in the United States. The remaining author declares that the research was conducted in the absence of any commercial or financial relationships that could be construed as a potential conflict of interest.

## Footnotes

^1^LMICs are defined by the World Bank here: https://datahelpdesk.worldbank.org/knowledgebase/articles/906519-world-bank-country-and-lending-groups

^2^CSOs exist in all countries as a “third sector” found between organizations that are public sector government and private sector commercial companies. NGOs and CSOs are often used interchangeably.

^3^Transparency International has championed a call for good governance of health care organization across the globe, see: http://www.transparency.org.uk/publications/transparency-good-governance-in-global-health/

^4^The authors recognize that non-governmental and private sector health systems in low and middle income countries are playing a more significant role in health services delivery. Governing these organizations will also benefit from the principles and practices of this chapter. For insights into the growing role of private health services organizations, see the WHO paper: http://www.who.int/bulletin/volumes/91/3/12-110791.pdf

^5^See: https://www.isixsigma.com/industries/healthcare/six-sigma-powerful-strategy-healthcare-providers/

^6^Correspondence with Mahesh Shukla, Management Sciences for Health, and meetings with the African Union in Addis Abba Ethiopia, Summer 2015.

^7^Health systems are defined by the World Health Organization as.…See: http://www.who.int/topics/

^8^For a comprehensive review of literature on governance in health, see: http://projects.msh.org/global-presence/upload/4-GovernanceLitReviewMay2012FINAL_PRINT.pdf

^9^USAID Health systems strengthening policy framework, see: https://www.usaid.gov/sites/default/files/documents/1864/HSS-Vision.pdf

^10^See: https://www.msh.org/resources/managers-who-lead-a-handbook-for-improving-health-services

^11^To see a comprehensive set of governance policies and guides, see: http://www.theglobalfund.org/en/governance/

^12^WHO insights into governance for health: http://www.who.int/healthsystems/publications/nhpsp-handbook/en/ and also http://www.who.int/universal_health_coverage/plan_action-hsgov_uhc.pdf and also here: http://www.euro.who.int/__data/assets/pdf_file/0019/171334/RC62BD01-Governance-for-Health-Web.pdf

^13^Insights into Governance and Corruption: http://www1.worldbank.org/publicsector/anticorrupt/Corruption%20WP_78.pdf

^14^OECD Health system governance http://www.oecd.org/els/health-systems/governance-health-systems.htm

^15^Competency based governance in US hospitals: http://trustees.aha.org/boardeval/10-competency-based-governance.pdf

^16^See: https://sustainabledevelopment.un.org/sdg3

